# High-Sensitivity and Anti-Interference Curvature Sensor Based on Optical Intensity Differential in Tapered Seven-Core Fiber

**DOI:** 10.3390/s26113365

**Published:** 2026-05-26

**Authors:** Jingshan Jia, Shuyang Duan, Meina Wu

**Affiliations:** School of Information and Communication Engineering, Xi’an University of Science and Technology, Xi’an 710054, China; 24207035002@stu.xust.edu.cn (S.D.); 25207223066@stu.xust.edu.cn (M.W.)

**Keywords:** fiber optic sensing, seven-core fiber, fused tapering, curvature sensing, differential sensing

## Abstract

**Highlights:**

**What are the main findings?**

- A tapered seven-core fiber (SCF) curvature sensor based on strong mode coupling is proposed and systematically investigated via numerical simulation.
- At an optimized taper waist diameter of 30 μm, the sensor achieves a high curvature sensitivity of −0.145/m^−1^ within the measurement range of 0–10 m^−1^.

**What are the implications of the main findings?**

- Taper-enhanced evanescent field coupling provides a robust mechanism for significantly increasing the sensitivity of multicore fiber platforms.
- The optical intensity differential ratio (ODR) scheme effectively eliminates common-mode interferences, such as light source fluctuations and environmental noise.
- High linearity and a compact architecture offer a stable and cost-effective solution for structural health monitoring in complex engineering environments.

**Abstract:**

This paper presents a high-sensitivity and anti-interference optical intensity differential curvature sensor based on seven-core fiber (SCF), and its performance is verified through simulation. The sensor adopts a “single-mode fiber–tapered SCF” structure, using the light intensity ratio between the peripheral core and the central core for curvature demodulation. It enhances the sensitivity while effectively suppressing common-mode interference. The simulation results show that when the cone zone diameter of the tapered SCF is optimized to 30 µm, the mode coupling between the cores is significant, forming a strongly coupled super-mode transmission system. Based on the intensity differential principle, this sensor achieves excellent linear response within the curvature range of 0–10 m^−1^, with a sensitivity of −0.145/m^−1^. The sensor has a compact structure and simple fabrication process, providing new ideas and solutions to break through the technical bottlenecks of existing fiber curvature sensors, and has broad application prospects in engineering monitoring fields.

## 1. Introduction

As a core physical quantity representing the deformation of an object, curvature has significant application value in fields such as aerospace strain mode monitoring, flexible robot motion control, and biomedical prosthetic feedback [1,2]. Due to their inherent advantages, such as a compact structure, fiber optic sensors have become a mainstream solution for curvature measurement [3]. Current common fiber optic curvature sensors are mainly divided into three types: fiber grating, interference, and optical modulation types. Among them, fiber grating-type curvature sensors usually require processes such as ultraviolet exposure or femtosecond laser writing [4], and their manufacturing process is relatively complex; interference-type curvature sensors, despite having high sensitivity [5], rely on expensive optical spectrum analyzers (OSAs) and photonic crystal fibers (PCFs) and are difficult to integrate due to the high cost of these special fibers; while despite having a simple structure and being low cost, optical modulation-type sensors generally have significant nonlinear effects and low sensitivity, and are susceptible to fluctuations in the light source and temperature crosstalk [6,7]. Multi-core fibers (MCFs) are widely used in curvature sensor design due to their unique spatial structural advantages [8], which can effectively improve the sensitivity of the sensor [9,10].

In recent years, there has been a growing number of curvature sensor designs based on multi-core optical fibers. In 2019, Zhang et al. proposed aligning the two off-center cores of a three-core fiber (TCF) with the two air holes of a double-sided-hole fiber (DSHF) to form a modal interferometer (MI) curvature sensor structure. By utilizing the minute refractive index difference between the two off-center cores to generate a cursor effect, they enhanced the sensitivity; within a curvature range of 0.5–1.2 m^−1^, sensitivity reached −60.84 nm/m^−1^ [11]. In 2020, Villatoro et al. designed a curvature sensor consisting of two asymmetric three-core fiber segments fused to a single-mode fiber; within a curvature range of 0 to −0.159 m^−1^, the sensitivity was 0.791 nm/m^−1^ [12]. In 2023, Zhu et al. proposed a curvature sensor combining a tapered seven-core fiber (SCF) with a peanut-shaped structure. Although this structure enhanced intermodal interference, it increased manufacturing complexity; its maximum sensitivity within the curvature range of 0 to 0.052 m^−1^ was 93.3374 nm/m^−1^ [13]. Leveraging their spatial structural advantages, multi-core optical fibers can effectively improve sensor sensitivity; however, existing designs may suffer from complex fabrication processes.

In this paper, we present a curvature sensor based on a single-mode fiber–tapered SCF heterogeneous fusion-spliced structure. By combining the high sensitivity of multimode fiber with the structural simplicity of an optical intensity modulation scheme, we employ the optical intensity differential as the demodulation method. The inter-core mode coupling is enhanced by optimizing the taper parameters; curvature demodulation is achieved using the optical intensity ratio between the outer and central cores, and common-mode interference caused by variations in temperature and optical intensity is canceled out by the differential changes in their optical intensities. Finite element simulations are used to investigate the light field propagation, bending strain, and optical power output characteristics of the taper region in tapered optical fibers. The results indicate that when the taper diameter is 30 μm, significant mode coupling and power redistribution occur in the fiber; within a curvature range of 0–10 m^−1^, the simulation results demonstrate an excellent linear response with a sensitivity of −0.145/m^−1^. This provides an effective solution to address the inherent limitations of optical intensity modulation sensors, such as the low sensitivity and weak interference resistance in multi-core fiber sensors.

## 2. Sensing Principle

**Figure 1:**
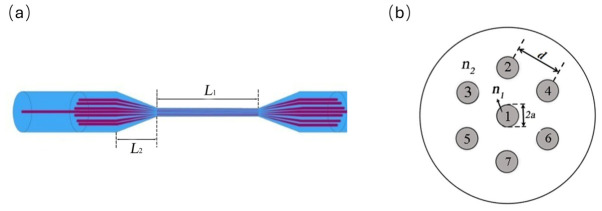
(**a**) Sensor structure diagram; (**b**) schematic diagram of the seven-core fiber cross-section.

Figure 1a shows a schematic diagram of the curvature sensor, fabricated by fusing a single-mode fiber to a tapered seven-core fiber. The waist region of the seven-core fiber has a length *L*_1_ of 9 mm, the transition region has a length *L*_2_ of 7 mm, and the waist region has a diameter of 30 µm. Figure 1b shows a schematic cross-sectional view of the seven-core optical fiber (core numbers 1, 2, …, 7). The cladding diameter is 240 µm, and all cores have a diameter of 9.8 µm (2a). The distance d between adjacent cores is 80 µm, and the refractive indices of the core and cladding, *n*_1_ and *n*_2_, are 1.461 and 1.444, respectively. Specifically, the geometric parameters of the untapered SCF region are precisely modeled to be consistent with the physical specifications of the commercial fibers utilized in our laboratory, thereby ensuring the experimental relevance and practical alignment of the numerical results. Finally, the output light from different cores is obtained via a seven-core fiber fan-in/fan-out module.

The amplitude evolution equations for the central and outer fibers of a seven-core optical fiber without taper are given by [14]

(1) $$A_{1}(z)=\left[\cos(Cz)+i\frac{K+\delta }{C}\sin(Cz)\right]e^{-i\phi_{0}z}$$

(2) $$A_{n}(z)=-i\frac{\kappa_{12}}{C}\sin(Cz)e^{-i\phi_{0}z}$$

where *C* $=\;\sqrt{{\left(K\;+\;\delta \right)}^{2}\;+\;6\kappa_{12}^{2}}$, *κ*_12_ represents the mutual coupling coefficient between the central and outer cores, *K* denotes the total coupling coefficient among the outer cores, and *δ* is the self-coupling coefficient. $\phi_{0}=K\;+\;\left(M_{1}\;+{\;M}_{2}\right)/2$ indicates the common phase factor. *M*_1_ and *M*_2_ are the self-coupling coefficients of the central and outer cores. The light intensity within the fiber core is *I = |A|*^2^. Assuming *I*_0_ is the initial input light intensity of the central core, substituting it into Equations (1) and (2) yields [15]

(3) $$\begin{array}{l} I_{1}(z)=I_{0}{\left|\cos(Cz)+i\frac{K+\delta }{C}\sin(Cz)\right|}^{2}\\ =I_{0}\left[\cos^{2}(Cz)+{\left(\frac{K+\delta }{C}\right)}^{2}\sin^{2}(Cz)\right] \end{array}$$

(4) $$\begin{array}{ll} I_{n}(z) & =I_{0}{\left|-i\frac{\kappa_{12}}{C}\sin(Cz)\right|}^{2}\\ & =I_{0}\left[{\left(\frac{\kappa_{12}}{C}\right)}^{2}\sin^{2}(Cz)\right] \end{array}$$

After undergoing the fusion tapering process, the optical fiber meets the strong coupling condition ($\kappa_{12}\;\gg \;K,\delta$), so *C*$\;\approx \;\sqrt{6}\kappa_{12}$, (K + δ)/C ≈ 0 Thus, Equations (3) and (4) can be reduced to:

(5) $$I_{1}\approx I_{0}\cos^{2}\left(\sqrt{6}\kappa_{12}L_{1}\right)$$

(6) $$I_{n}\approx \frac{1}{6}I_{0}\sin^{2}\left(\sqrt{6}\kappa_{12}L_{1}\right)$$

Here, *L*_1_ is the waist length of the tapered region. According to the multi-core fiber mode coupling theory, the coupling coefficient *κ*_12_ is related to the refractive index difference, and its change Δ*κ* satisfies [16]

(7) Δκ∝Δn

Under bending, the strain-induced refractive index change Δ*n_r_* caused by the elasto-optic effect is given by [17]:

Upon sensing, the refractive index change Δ*n_r_* induced by the strain *ε* via the elasto-optic effect is given by

(8) $$\Delta n_{r}=-\frac{1}{2}n_{0}^{3}\left[p_{12}-\nu \left(p_{11}+p_{12}\right)\varepsilon \right]$$

In this equation, *p*_11_ and *p*_12_ are the elastic refractive indices, *ν* is Poisson’s ratio, and *ε* = *y/R* is the axial strain caused by bending, where *y* is the distance from the core to the neutral axis, and *R* is the radius of curvature. When the sensor is bent, the elastic-optical effect causes a change in the fiber cross-section refractive index, which in turn leads to a change in the inter-mode coupling coefficient Δ*κ_R_*, ultimately resulting in a redistribution of the light intensity output from each core. It is worth noting that although bending also causes a slight deformation of the core-to-core spacing, its effect on the coupling coefficient is far smaller than the dominant effect of the change in refractive index [18].

In practical applications, the thermo-optic effect caused by temperature fluctuations also alters the effective refractive index, and the magnitude of this change is given by [17]

(9) $$\Delta n_{\mathrm{eff},T}=\frac{dn_{\mathrm{eff}}}{dT}\cdot \Delta T$$

Here, $\frac{dn_{\mathrm{eff}}}{dT}$ represents the thermo-optic coefficient, and Δ*T* denotes the change in temperature. This change in refractive index further causes a change in the coupling coefficient between the central and outer core, denoted as Δ*κ_T_*, which interacts with the change in the coupling coefficient Δ*κ_R_* induced by bending strain to influence the light field propagation process. When the sensor is subjected to both bending deformation and temperature fluctuations, the output light intensity of the central and outer core can be expressed as

(10) $$I_{1}^{\prime }=I_{0}\cos^{2}\left[\sqrt{6}\left(\kappa_{12}+\Delta \kappa_{R}+\Delta \kappa_{T}\right)L_{1}\right]$$

(11) $$I_{n}^{\prime }=\frac{1}{6}I_{0}\sin^{2}\left[\sqrt{6}\left(\kappa_{12}+\Delta \kappa_{R}+\Delta \kappa_{T}\right)L_{1}\right]$$

Here, *I*_0_ represents the initial input light intensity. When bending and temperature fields coexist, the output light intensity of a single fiber core is affected by the combined influence of both, and is highly susceptible to fluctuations in the light source power *I*_0_ and link loss, leading to measurement errors. To address this issue, differential demodulation is performed using the light intensity ratio between the central and peripheral cores. Substituting the above light intensity expressions yields

(12) $$r=6\cot^{2}\left[\sqrt{6}\left(\kappa_{12}+\Delta \kappa_{R}+\Delta \kappa_{T}\right)L_{1}\right]$$

As shown in Equation (12), the expression for the ratio r no longer includes the initial light intensity *I*_0_, thereby fundamentally eliminating the additive noise introduced by light source instability and fluctuations in transmission loss. Furthermore, since the effect of temperature changes on the refractive indices of the individual fiber cores exhibits a consistent spatial distribution, the resulting coupling coefficient variation can be regarded as a common-mode signal. By performing ratio calculations on the light intensities, effective common-mode rejection can be achieved, enabling high-precision curvature measurement without the need for additional temperature compensation modules.

## 3. Simulation and Analysis

### 3.1. Simulation of the Light Field in Tapered Optical Fibers

**Figure 2:**
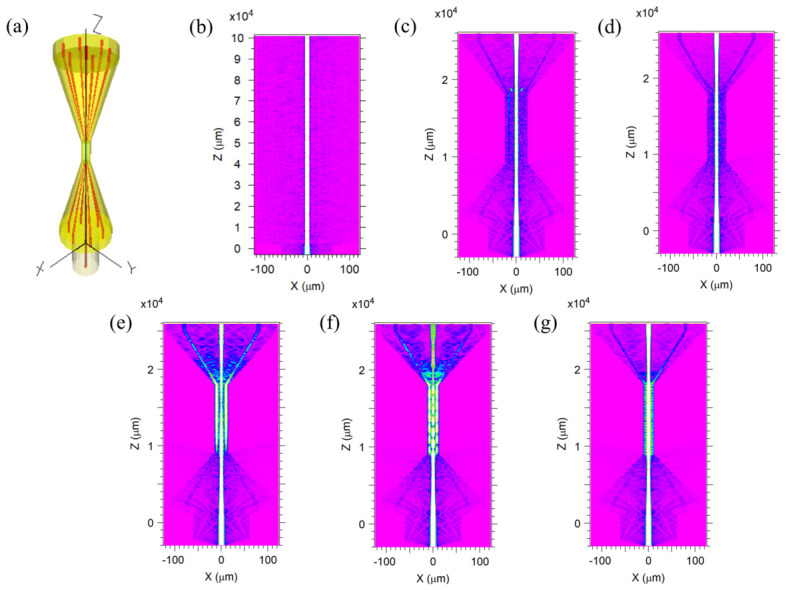
(**a**) Three-dimensional model of tapered seven-core optical fiber in Rsoft; (**b**) optical field distribution of seven-core fiber without tapering. (**c**) Light field distributions when cone diameter is 48 µm; (**d**) 40 µm; (**e**) 30 µm; (**f**) 24 µm; and (**g**) 20 µm.

To investigate the effect of tapered structures on the light field distribution in seven-core optical fibers, in this study, we used Rsoft software 8.0 to construct a three-dimensional model based on the beam propagation method (BPM), focusing on the taper diameter’s influence on light field evolution. A total of six simulation scenarios were established with different taper diameters, and the 3D model of a tapered seven-core fiber constructed in Rsoft is shown in Figure 2a (see Table 1 for the single-mode and untapered seven-core fiber parameters); Figure 2b shows the steady-state optical field of the seven-core fiber without tapering. At this point, the core-to-core spacing is 80 μm, and there is almost no mode coupling between the cores; the optical field energy is primarily confined to the central core, with almost no cladding modes generated. The steady-state optical fields for taper diameters of 48 μm and 40 μm are shown in Figure 2c and Figure 2d, respectively. Cladding modes gradually emerge, but due to the large core-to-core spacing, the power redistribution effect is not significant. The steady-state light fields for cone diameters of 30 μm and 24 μm are shown in Figure 2e,f; at this point, it can be seen that the gradual reduction in core spacing further enhances coupling, resulting in significant and stable mode coupling. Energy is effectively transferred between the central and peripheral cores, forming a strongly coupled supermode transmission system, conditions under which the fiber is well-suited for use as a curvature sensor. Clearly, when the taper diameter is 30 μm, the mode in the core is more stable, and the peripheral core output intensity remains essentially the same as that of the core mode. The steady-state light field when the taper diameter is 20 μm is shown in Figure 2g. Excessive tapering causes distortion of the core structure, resulting in significant inter-mode coupling. The inter-core coupling degree decreases, making it unsuitable for use as a sensor; therefore, the best inter-core coupling effects are achieved when the taper diameter is 30 μm and 24 μm. The drawing results for these two conditions were selected for bending strain simulation.

### 3.2. Sensor Simulation and Analysis

#### 3.2.1. Model Development and Light Output

**Figure 3:**
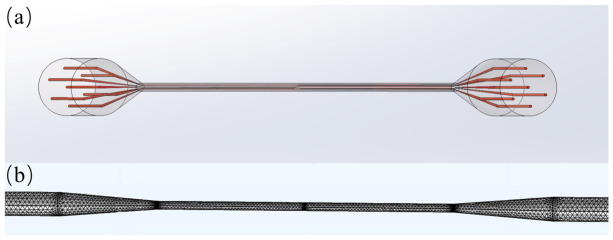
(**a**) Three-dimensional model of tapered seven-core optical fiber in SolidWorks 2024; (**b**) free triangular mesh of the tapered seven-core fiber three-dimensional model in COMSOL 6.3.

To verify the validity of the light intensity differential curvature sensor theoretical model proposed in this paper, we first used SolidWorks 3D modeling software (2024) to construct a tapered optical fiber model with precise geometric features, as shown in Figure 3a. For the core influencing parameter—the taper region diameter—six sets of variables were set: 80 μm, 48 μm, 40 μm, 30 μm, 24 μm, and 20 μm, while the transition and waist zone lengths were fixed at 7 mm and 9 mm, respectively (see Table 1 for other relevant structural parameters). The geometric accuracy of the model was strictly controlled at the submicron level to lay the foundation for the accuracy of subsequent simulation analyses.

To accurately simulate the evolution of the light field under fiber bending conditions and obtain the output light intensities at the center and periphery of the fiber core, optical propagation simulations and numerical analyses were conducted using the COMSOL 6.3 multiphysics simulation platform. A 3D model was imported into the COMSOL environment, and electromagnetic field analysis was performed using the frequency-domain solver in the “Wave Optics” module.

For material property settings, the effective refractive index of the core, n_1_, is set to 1.461, and that of the cladding, n_2_, is set to 1.444. The operating wavelength for the simulation is set to 1550 nm. The structural mesh is defined using a physically field-controlled free triangular mesh. As shown in Figure 3b, the mesh is refined in regions where the optical field intensity varies (such as the core–cladding interface). The minimum element size is set to 0.1 μm, and the maximum element growth rate is 1.2, ensuring that the mesh quality Jacobian matrix condition number is less than 3. To obtain the sensor output characteristics of the system, an electromagnetic field monitoring plane is positioned at the output end face of the seven-core fiber to accurately capture the two-dimensional light intensity distribution and the independent output light intensity data for each core.

To maintain numerical accuracy in the bending simulation, a 5 μm thick Perfectly Matched Layer (PML) combined with Scattering Boundary Conditions (SBCs) is implemented. This configuration ensures that the radiation field energy is effectively absorbed, preventing non-physical reflections from interfering with the inter-core mode coupling and curvature response. This ensures that the far-field radiation conditions align with those of the actual scenario, thereby reducing errors. To ensure the reliability of the results, five independent simulations are performed for each set of parameters within a curvature range of 0 to 10 m^−1^, with a step size of 1 m^−1^. The consistency of the repeated simulation results verifies the stability of the model and provides a reliable data foundation for subsequent analysis.

#### 3.2.2. Bending Simulation and Analysis of Results

When performing bending simulations, an equivalent model incorporating a bending refractive index distribution is introduced. The beam propagation method is employed, combined with conformal mapping techniques, to simulate bent optical fibers. By varying the fiber’s effective refractive index, the simulation captures the changes in output caused by bending strain during light propagation within the fiber. Conformal mapping is used to transform a circular bent fiber into an equivalent straight fiber [17], and the expression for the bending equivalent model is [18]

(13) $$n^{\prime }=n\left(1+\frac{x}{R_{\mathrm{eff}}}\right)$$

**Figure 4:**
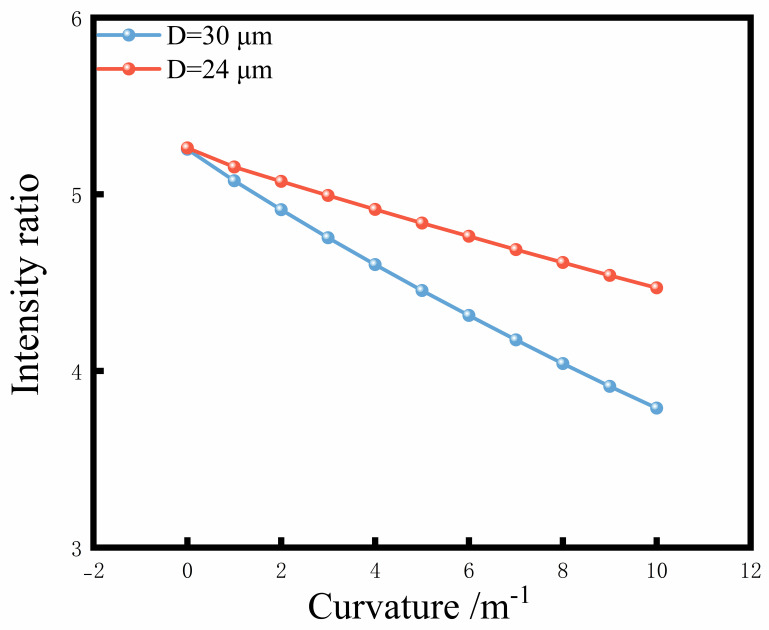
Variation in the light intensity ratio between the two fiber cores with curvature/m^−1^ when the taper diameter is 30 μm and 24 μm.

Here, Reff represents the radius of curvature. As shown in Figure 1b, the No. 2 core of the tapered seven-core fiber was bent, and its light intensity and that of the central core were measured in 1 m^−1^ increments over a curvature range of 0–10 m^−1^. The simulation results are shown in Figure 4. As can be seen from the figure, the light intensity ratio between the two cores varies with curvature when the taper diameters are 30 μm and 24 μm, respectively; as the curvature gradually increases, the light intensity ratio decreases significantly under both taper diameter conditions, with the 30 μm taper diameter scheme exhibiting a more pronounced rate of decrease, demonstrating superior curvature response sensitivity.

When the taper diameter is reduced to 24 μm, the inter-mode coupling effect increases significantly due to the overly compact fiber structure, resulting in a substantial attenuation of the output light intensity from the central core, while, in contrast, the attenuation of that from the outer core is relatively minor. Consequently, the reduction in the light intensity ratio between the two cores is relatively small, leading to a curvature response sensitivity that is lower than that of the 30 μm taper diameter design.

**Figure 5:**
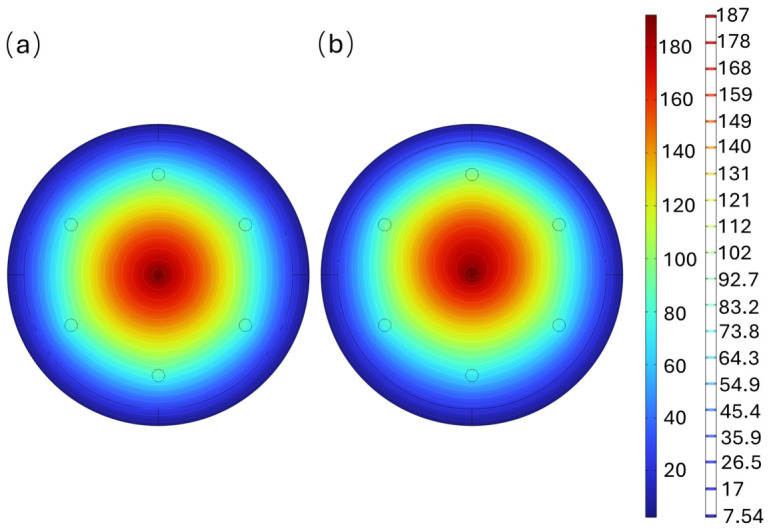
Simulated electric field mode distributions of the tapered SCF under bending curvatures of (**a**) *C*= 1 m^−1^ and (**b**) *C* = 10 m^−1^. The white lines represent the electric field mode contours.

**Figure 6:**
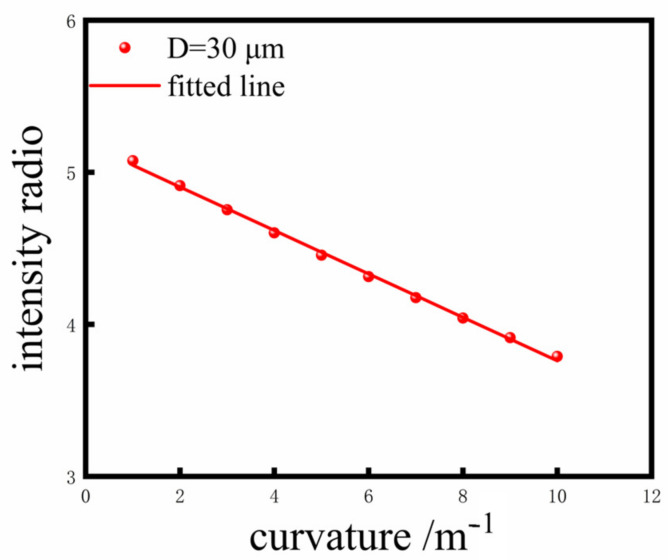
Data fitting of the variation in the light intensity ratio between the two fiber cores with curvature when the taper diameter D = 30 μm.

To accurately simulate the variation in the intensity ratio as a function of curvature for a conical region with a diameter of 30 μm, intensity distribution contour plots were generated for different curvatures using an electromagnetic field monitoring plane. The results are shown in Figure 5; specifically, Figure 5a,b correspond to the intensity distribution characteristics at curvatures of 1 m^−1^ and 10 m^−1^, respectively. As can be clearly observed from the figure, as the curvature increases, the light intensity distribution shifts in the direction opposite to the bend. To clearly illustrate the mode coupling dynamics, white contour lines representing the optical intensity distribution are superimposed in Figure 5. As C increases from 1 m^−1^ to 10 m^−1^, the optical power, indicated by the continuous compression of the contours, significantly shifts from the central core to core 2. This directly verifies that the bend-induced refractive index gradient dominates the enhanced evanescent field coupling. Furthermore, based on the aforementioned intensity distribution contour plots, the average output light intensity within the corresponding core regions was extracted and processed. The resulting relationship between the light intensity ratio of the two cores and curvature, along with the fitting results, is shown in Figure 6. The fitting equation is *y* = −0.145*x* + 5.216. Here, *y* represents the ratio of light intensity between the central core and core 2, and x represents the curvature. The fitting results show that the coefficient of determination (R^2^) reaches as high as 0.997, and the Pearson correlation coefficient is −0.999, fully confirming the significant negative linear correlation between the light intensity ratio and curvature, and indicating that within a curvature range of 0 to 10 m^−1^, the sensitivity of this sensor is −0.145/m^−1^. The sensor design demonstrates excellent stability and repeatability, and its practical value has been effectively validated.

#### 3.2.3. Manufacturing Tolerance Analysis

**Figure 7:**
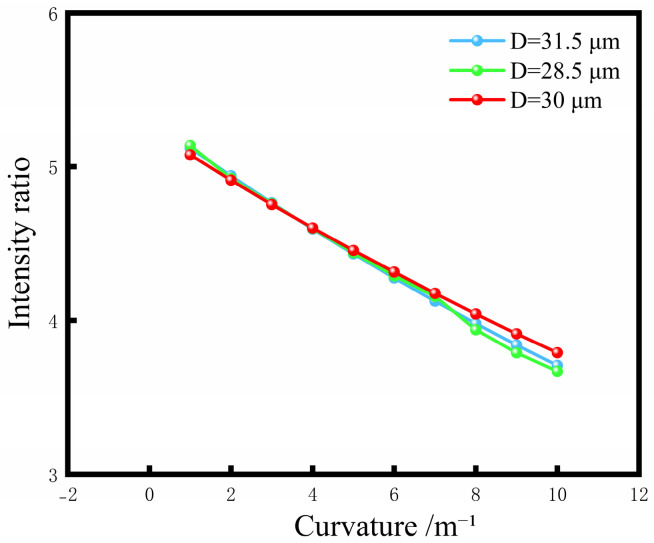
Manufacturing tolerance analysis of the optical intensity differential ratio output under different taper waist diameters.

In practical fabrication, dimensional deviations of the tapered SCF are inevitable due to the precision limits of micro-nano drawing processes. To evaluate structural robustness, the influence of manufacturing tolerance is analyzed by varying the taper waist diameter by ±5% (28.5 μm and 31.5 μm) around the optimal 30 μm. As shown in Figure 7, the proposed optical intensity differential scheme maintains a stable and monotonic linear response despite these geometric fluctuations. This indicates that the measurement technique exhibits high tolerance to manufacturing errors, ensuring the reliability of the sensor.

#### 3.2.4. Full-Space Directional Bending Response

**Figure 8:**
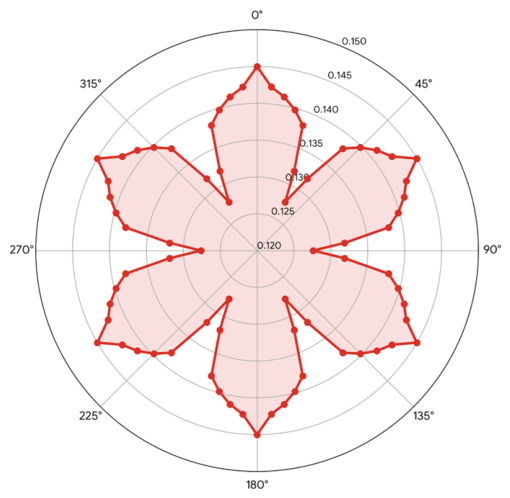
Full-space sensitivity boundary radar chart of the tapered seven-core optical fiber sensor.

Furthermore, the bending direction in actual 3D structural health monitoring is typically random. Based on the 60° rotational symmetry of the proposed SCF, an angular response analysis is performed within the irreducible symmetric interval of 0° to 30° with a step size of 5°. The extracted sensitivities are utilized to reconstruct a 360° full-space sensitivity boundary radar chart, as presented in Figure 8. The maximum and minimum sensitivities are −0.145 m^−1^ (at 0°) and −0.1276 m^−1^ (at 30°), respectively. The sensor maintains >88% of its peak sensitivity across the entire 360° range with high linearity (*R*^2^ > 0.998), demonstrating excellent isotropic adaptability for arbitrary bending directions.

### 3.3. Temperature Response Simulation and Analysis

**Figure 9:**
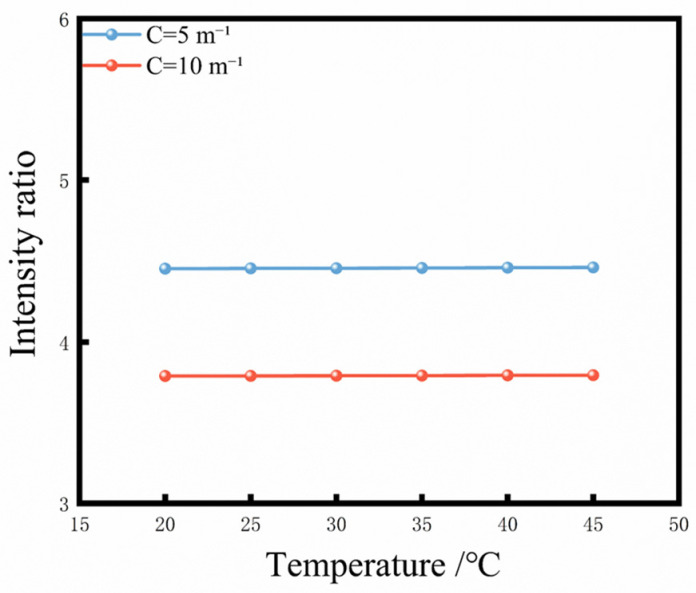
Sensor output characteristics under different temperatures.

To further verify the sensor’s immunity to interference in real-world applications, and specifically to evaluate the effectiveness of the optical intensity differential demodulation scheme in suppressing temperature crosstalk, in this study we conducted sensor temperature response simulations. Based on the theory of the thermo-optic effect (Equation (9)), temperature changes cause alterations in the refractive index of the optical fiber material. In the simulation, the ambient temperature range was set from 20 °C to 45 °C, with a step size of 5 °C, during which the sensor was maintained in two fixed states with curvatures of 5 m^−1^ and 10 m^−1^, and the changes in the output light intensity ratio between the central and outer core (core 2) were recorded. The results are shown in Figure 9.

As shown in Figure 9, under different curvature conditions, the light intensity ratio between the central and outer core remains essentially stable as the temperature rises, and the curve exhibits excellent flatness. The extracted temperature sensitivities are extremely low, calculated as 3.0 × 10^−4^/°C for C = 5 m^−1^ and 2.06 × 10^−4^/°C for C = 10 m^−1^, both exhibiting high linearity (*R*^2^ = 0.999).

Although the thermo-optic effect causes slight drifts in the refractive indices of the core and cladding, which in turn leads to changes in the coupling coefficient *κ*_12_, the seven-core fiber consists of cores with identical materials and a compact spatial distribution. Therefore, changes in the temperature field can be regarded as a common-mode signal acting uniformly on all cores. Through the ratio operation defined by Equation (12), the temperature-induced fluctuations in light intensity in the same direction in the numerator and denominator cancel each other out. Simulation data indicate that within this temperature range, the standard deviation of the intensity ratio is extremely small, and the measurement error introduced by temperature is negligible. This result strongly demonstrates that the proposed differential demodulation method effectively overcomes the weakness of traditional intensity-modulation-type sensors, which are susceptible to temperature crosstalk, thereby achieving temperature self-compensation in curvature measurement.

## 4. Conclusions

In this paper, we propose a tapered seven-core optical fiber curvature sensor and validate it through simulation. The sensor consists solely of a single-mode and a seven-core optical fiber. The fabrication process is as follows: First, the single-mode fiber is core-to-core fused with a section of the seven-core fiber. Next, inter-core coupling is enhanced through a fusion-tapping process. Finally, the fan-in/fan-out module is used to collect the output optical intensity of each core, and the fusion-tapping method is employed to generate strong inter-core coupling modes, allowing the output optical intensity of each core to be directly obtained. By monitoring the optical power ratio between the outer fibers (such as fiber 2) and the central fiber, bending curvature can be detected, thereby enhancing sensitivity and suppressing common-mode interference. Using Rsoft and COMSOL software, we investigated the light field and output characteristics of the sensor after conical stretching by simulating the sensor under various parameters and performing bending simulations to obtain optimized parameters and high-linearity response characteristics. The results indicate that, at the optimal taper diameter (30 µm), the sensor exhibits extremely high linearity (*R*^2^ = 0.997) and sensitivity (−0.145/m^−1^) within the range of 0–10 m^−1^. This design features a simple structure and requires no complex spectral demodulation equipment. Combining high sensitivity with strong anti-interference capabilities, it provides a low-cost, high-performance solution for monitoring structural deformation in precision engineering.

While in this study we focus on numerical analysis, fabricating the proposed tapered SCF is highly feasible. Commercial SCFs can be precisely tapered to the optimal 30 μm waist diameter using mature techniques, such as the flame brushing method or CO_2_ laser heating. The optimized parameters presented herein provide crucial guidance for actual device fabrication, minimizing experimental trial and error. Future work will focus on the physical implementation, experimental calibration, and packaging of the sensor for practical structural health monitoring applications.

Finally, it is worth noting that in this study we present a proof-of-concept theoretical demonstration. The physical fabrication of the proposed sensor requires high-precision heterogeneous fusion splicing (SMF-to-SCF) and submicron-scale tapering, posing challenges for current manufacturing platforms. Nevertheless, the comprehensive tolerance and full-space directional analyses validate the feasibility and robustness of the design. In future work, we will focus on the physical implementation and experimental calibration of the sensor system.

## Figures and Tables

**Table 1:** Parameters of the single-mode fiber and the multi-core fiber (before tapering).

Type	Core Radius	Cladding Radius	Core Refractive Index	Cladding Refractive Index	Core Pitch
Single-mode fiber	4.5 µm	62.5 µm	1.449	1.444	—
Multi-core fiber	4.9 µm	120 µm	1.461	1.444	80 µm

## Data Availability

The raw data supporting the conclusions of this article will be made available by the authors on request.

## Abbreviations

The following abbreviations are used in this manuscript:

SCF	Seven-core Fiber
OSA	Optical Spectrum Analyzer
PCF	Photonic Crystal Fiber
MCF	Multi-core Fiber
TCF	Three-core Fiber
DSHF	Double-sided-hole Fiber
MI	Modal Interferometer
